# Supplementary material for: Self-Compatibility Not Associated with Morphological or Genetic Diversity Reduction in Oil-Rewarding Calceolaria Species
Source: Plants (Basel). 2020 Oct 16;9(10):1377. doi: 10.3390/plants9101377 (PMC7602863; doi:10.3390/plants9101377)

Supplementary Material

Table S1. Genetic parameters of nine SSR markers for nine *Calceolaria* species under study. Number of alleles per locus (*Na*), observed (*Ho*) and expected (*He*) heterozygosity, and the fixation index (F_{IS}) are informed. Values of Mean ± Standard Error are showed for all markers by species.

	C. arachnoidea				*C. filicaulis spp. filicaulis*				*C. filicaulis spp. luxurians*			
**Locus**	**Na**	**Ho**	**He**	**Fis**	**Na**	**Ho**	**He**	**Fis**	**Na**	**Ho**	**He**	**Fis**
618	2.000	0.000	0.320	1.000	2.000	0.000	0.180	1.000	4.000	0.133	0.736	0.819
693	5.000	0.500	0.530	0.057	2.000	0.000	0.500	1.000	4.000	0.167	0.521	0.680
2778	7.000	0.600	0.790	0.241	5.000	0.375	0.727	0.484	9.000	0.667	0.822	0.189
610	1.000	0.000	0.000	NA	2.000	0.000	0.180	1.000	5.000	0.071	0.691	0.419
1649	2.000	0.000	0.278	1.000	1.000	0.000	0.000	NA	4.000	0.200	0.344	0.101
1777	6.000	0.800	0.755	-0.060	5.000	0.400	0.585	0.316	11.000	0.733	0.816	0.402
1273	2.000	1.000	0.500	-1.000	5.000	0.700	0.585	-0.197	5.000	0.308	0.515	0.679
1540	7.000	0.143	0.827	0.827	2.000	0.000	0.198	1.000	11.000	0.267	0.831	0.438
2075	2.000	0.000	0.180	1.000	3.000	0.125	0.227	0.448	7.000	0.308	0.547	0.897
**Mean**	**3.778**	**0.338**	**0.464**	**0.383**	**3.000**	**0.178**	**0.353**	**0.631**	**6.667**	**0.317**	**0.647**	**0.514**
**SE**	**0.813**	**0.131**	**0.097**	**0.238**	**0.527**	**0.085**	**0.083**	**0.148**	**0.986**	**0.077**	**0.057**	**0.091**

	C. integrifolia				*C. lanigera*				*C. petiolaris*			
**Locus**	**Na**	**Ho**	**He**	**Fis**	**Na**	**Ho**	**He**	**Fis**	**Na**	**Ho**	**He**	**Fis**
618	2.000	0.100	0.255	0.608	3.000	0.000	0.582	1.000	2.000	0.000	0.320	1.000
693	4.000	0.333	0.543	0.386	6.000	0.533	0.551	0.032	3.000	0.167	0.542	0.692
2778	5.000	0.111	0.710	0.843	6.000	0.286	0.518	0.448	3.000	0.500	0.485	-0.031
610	1.000	0.000	0.000	NA	5.000	0.077	0.642	0.880	1.000	0.000	0.000	NA
1649	2.000	0.100	0.095	-0.053	7.000	0.500	0.518	0.034	3.000	0.000	0.580	1.000
1777	2.000	0.300	0.255	-0.176	3.000	0.357	0.523	0.317	1.000	0.000	0.000	NA
1273	4.000	0.250	0.414	0.396	2.000	0.846	0.488	-0.733	2.000	0.300	0.255	-0.176
1540	3.000	0.500	0.615	0.187	6.000	0.250	0.479	0.478	4.000	0.000	0.640	1.000
2075	3.000	0.143	0.357	0.600	7.000	0.214	0.796	0.731	4.000	0.200	0.345	0.420
**Mean**	**2.889**	**0.204**	**0.360**	**0.349**	**5.000**	**0.340**	**0.566**	**0.354**	**2.556**	**0.130**	**0.352**	**0.558**
**SE**	**0.423**	**0.052**	**0.079**	**0.116**	**0.624**	**0.086**	**0.033**	**0.177**	**0.377**	**0.060**	**0.079**	**0.167**

Genebank accession number for each locus: Locus 618= MF574823; Locus 693= MF574828; Locus 2778=574820; Locus 610=574822; Locus 1649=MF574817; Locus 1777= MF574825; Locus 1273= MF574816; Locus 1540= MF 574825; Locus 2075= MF574819).

	C. polifolia				*C. purpurea*				*C. segethii*			
**Locus**	**Na**	**Ho**	**He**	**Fis**	**Na**	**Ho**	**He**	**Fis**	**Na**	**Ho**	**He**	**Fis**
618	3.000	0.100	0.265	0.623	4.000	0.500	0.410	-0.220	2.000	0.000	0.180	1.000
693	4.000	0.200	0.670	0.701	3.000	0.000	0.593	1.000	1.000	0.000	0.000	NA
2778	5.000	0.400	0.630	0.365	5.000	0.000	0.735	1.000	2.000	0.100	0.095	-0.053
610	3.000	0.600	0.445	-0.348	2.000	0.100	0.095	-0.053	2.000	0.000	0.278	1.000
1649	4.000	0.100	0.725	0.862	2.000	0.000	0.444	1.000	3.000	0.000	0.611	1.000
1777	4.000	0.100	0.695	0.856	4.000	0.222	0.574	0.613	2.000	0.000	0.180	1.000
1273	5.000	0.600	0.660	0.091	3.000	0.800	0.560	-0.429	3.000	0.000	0.540	1.000
1540	3.000	0.111	0.290	0.617	6.000	0.125	0.789	0.842	3.000	0.100	0.185	0.459
2075	3.000	0.600	0.555	-0.081	2.000	0.000	0.420	1.000	5.000	0.500	0.665	0.248
**Mean**	**3.778**	**0.312**	**0.548**	**0.410**	**3.444**	**0.194**	**0.513**	**0.528**	**2.556**	**0.078**	**0.304**	**0.707**
**SE**	**0.278**	**0.079**	**0.058**	**0.144**	**0.475**	**0.093**	**0.068**	**0.197**	**0.377**	**0.055**	**0.080**	**0.142**

Figure S1. Mid-rooted Neighbor-Joining (NJ) tree of the nine *Calceolaria* species based on distances of nine SSR markers. This figure comprises all individuals, including those with missing alleles. *Calceolaria* species: ARA: *C. arachnoidea*, FIL: *C. filicaulis* spp. *filicaulis*, FILUX: *C. filicaulis* spp. *luxurians,* INT: *C. integrifolia*, LAN: *C. lanigera,* PET: *C. petiolaris,* POL: *C. polifolia,* PUR: *C. purpurea,* and SEG: *C. segethii*. Scale bar indicates genetic distance. Numbers after species name indicate sample names. Bootstrap support values higher or equal to 0.7 are shown on nodes.

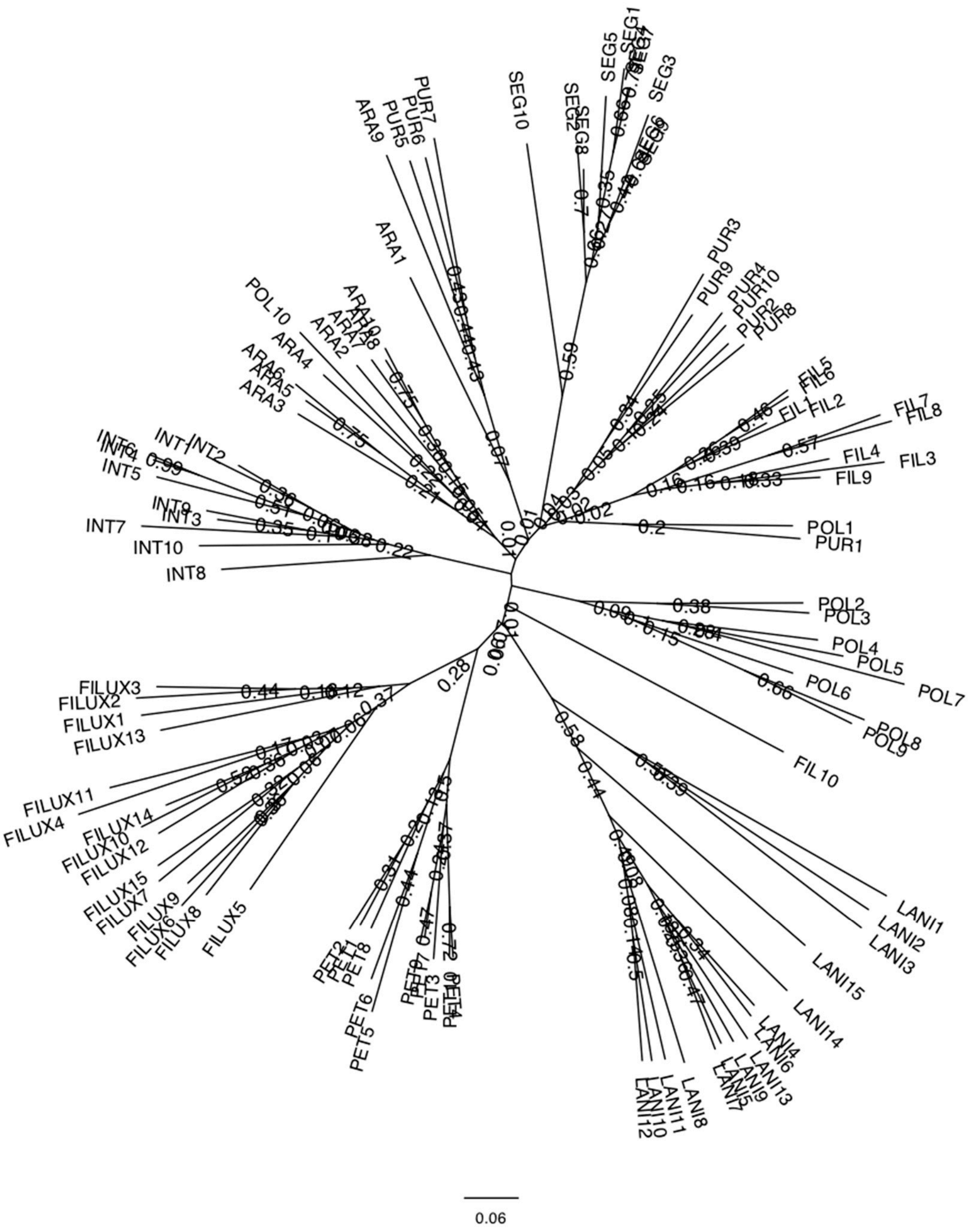

Supplement: Supplementary file 1 [file plants-09-01377-s001.pdf]
